# Towards an engineering theory of evolution

**DOI:** 10.1038/s41467-021-23573-3

**Published:** 2021-06-07

**Authors:** Simeon D. Castle, Claire S. Grierson, Thomas E. Gorochowski

**Affiliations:** 1 School of Biological Sciences, University of Bristol, Bristol, UK; 2 BrisSynBio, University of Bristol, Bristol, UK

**Keywords:** Biotechnology, Evolution, Evolutionary theory, Synthetic biology

## Abstract

Biological technologies are fundamentally unlike any other because biology evolves. Bioengineering therefore requires novel design methodologies with evolution at their core. Knowledge about evolution is currently applied to the design of biosystems ad hoc. Unless we have an engineering theory of evolution, we will neither be able to meet evolution’s potential as an engineering tool, nor understand or limit its unintended consequences for our biological designs. Here, we propose the evotype as a helpful concept for engineering the evolutionary potential of biosystems, or other self-adaptive technologies, potentially beyond the realm of biology.

## Abstract (web summary)

Effective biological engineering requires the acknowledgement of evolution and its consideration during the design process. In this perspective, the authors present the concept of the evotype to reason about and shape the evolutionary potential of natural and engineered biosystems.

## Introduction

The past few decades have seen a revolution in our ability to engineer biology and create living systems with novel functions^1^. Yet, several hurdles still hinder our capability to harness biology’s full potential^2^. These stem from the fact that you cannot engineer the stuff of life without engineering its properties too and life’s most fundamental property is that it evolves. Evolution makes engineering living systems a radically different challenge to engineering other mediums. To be effective, we cannot simply apply traditional engineering design principles to biology and deal with evolution as a secondary thought. If nothing in biology makes sense except in the light of evolution^3^, then evolution must be a central part of an engineering theory of biology.

Evolution poses both a challenge and an opportunity when designing biosystems. On one hand, it is a detrimental force that can unpick the meticulous plans of an engineer through genetic variation^4^. Designed biosystems cannot escape evolution when used and loss of function is a particular concern for engineers, especially as there are often selection pressures working against the design’s function^5,6^. It is essential that we learn to build evolutionarily stable biosystems that can continue to operate under unavoidable evolutionary forces.

On the other hand, evolution is an extremely effective problem solver and engineers have exploited this fact for decades^7–10^. For example, directed evolution can be used to optimise or even generate completely novel traits in proteins^8,11^ or cells^12^. However, these methods rely on the ability of evolution to find solutions in a reasonable length of time. For most systems, the search space is so vast that the starting point in this process must have the potential to generate useful phenotypes relatively quickly.

Evolution may even be employed as a feature of the system during operation. For example, adaptive systems that evolve in response to environmental cues or evolvable genetic circuits that can be designed with specific classes of phenotype that are reached as necessary through evolutionary change. To create such systems, it is critical that the biological design is specifically evolvable. This means it must have the potential to generate the types of phenotypes desired by the engineer from a single starting point in a reasonable time frame.

Even more critical is our moral obligation to develop a deeper understanding of how synthetic biosystems will continue to evolve if deployed into our bodies or the wider environment^13^. The field has rightly made efforts to develop tools to reduce and mitigate evolution^14^, with fail-safes such as kill switches^15^ or metabolic dependencies^16^. However, without a good theoretical understanding of how synthetic biosystems might continue to evolve once deployed, we risk these technologies developing unexpected faults with dire, but avoidable, consequences. Even breeding has at times had dire consequences. Notably, the inadvertent creation of the hyper-aggressive Africanised bee, which has had a severe impact on humans and ecology^17^. As we develop technologies capable of even more rapid genetic change, such as gene drives^18^, these concerns will become even more salient.

**Fig. 1:**
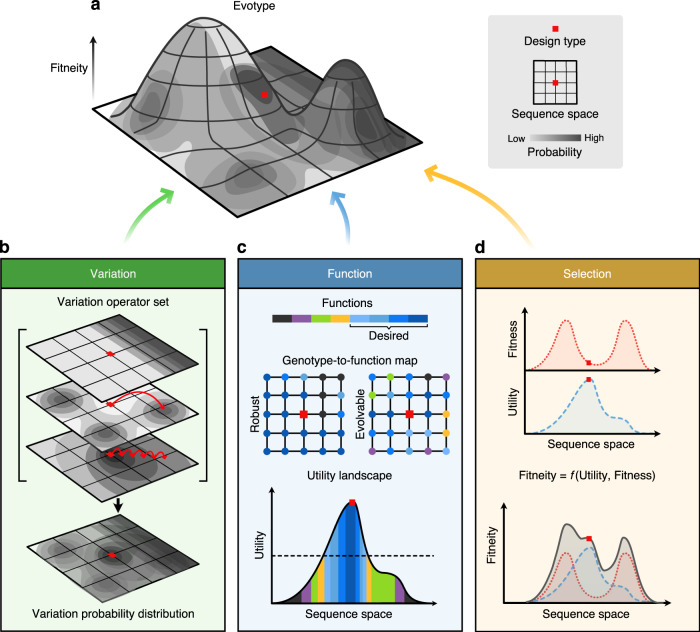
The evotype and its key properties. **a** The evotype visualised as a landscape surrounding the design type (red square), where fitneity (the combined function of fitness and utility) is plotted as a vertical axis against a 2D plane of sequence space with the probability of evolution exploring regions of sequence space overlaid in grey. The properties of this landscape are determined by the interaction of three components: variation, function and selection. **b** A variation probability distribution can be projected onto sequence space, which represents the likelihood of exploring a given sequence through genetic variation. Darker regions represent regions of higher probability. This is the sum of the distributions of the individual variation operators present in the system (variation operator set). For example, point mutation (bottom layer in set), recombination of homologous regions (middle layer in set), and slip-strand mutation (top layer in set). Red arrows in the middle and bottom layers represent algorithmic and point mutations, respectively. **c** How phenotypic functions are distributed in sequence space surrounding the design type is critical. Function space may be considered as discrete (top), where the space may have high genotypic robustness (left grid) or high variability (right grid). A continuous utility space (bottom) plotted against a 1D projection of sequence space. The colour under the curve represents the discrete function associated with that region of sequence space and the utility that each has as a continuous value. For example, if the goal is to produce blue-like functions, dark blue may have the highest utility, followed by lighter variants in the spectrum. The bioengineer must define a minimal threshold (dashed line), below which the design is deemed to be a failure (e.g., non-desired function is exhibited). **d** Sequences differ in their reproductive fitness. This is the driver of natural selection and can be plotted across sequence space as an adaptive landscape (red dotted line). Utility (blue dashed line) may or may not correlate with reproductive fitness across sequence space. The fitneity (grey solid line) is the combination of the fitness and utility. Bioengineers must optimise fitneity both for the design type and throughout the landscape.

Central to many of these issues is the view in traditional engineering disciplines that the engineered artefact is a final destination in the design process. This view breaks for biology. Instead, we believe that a new perspective is needed for a truly effective engineering of biology; one that sees a designed biosystem as a starting point in a lineage of possibilities. Although much of evolutionary biology has concerned itself with organisms’ histories^19^, bioengineers must consider the future and, specifically, how a biosystem will continue to evolve when used^20^. Here we describe a framework that enables this transition and offers a way to specify, test and conceive the properties of biosystems in terms of their evolutionary potential and not just their phenotype (Fig. 1). This provides a way to re-imagine biological engineering so that it works in harmony with life’s ability to evolve.

## The design type and the evotype

To better harness the capabilities of biology, we need a way of thinking about the evolutionary properties of engineered biosystems. We must design for potential evolutionary change and not just the immediate functionalities displayed by a system (i.e., its phenotypic traits). Although these are properties of populations yet to exist, they can still be considered in the context of an individual biosystem. We consider the ‘design type’ as a system that has been engineered, consisting of a single genotype. The design type could be any biosystem capable of evolution: a protein, genetic circuit, virus, cell, animal, plant or even an ecosystem. We introduce the concept of the ‘evotype’ to capture the evolutionary properties of that system. The evotype is a set of evolutionary dispositions of the design type, analogous to genotype and phenotype being sets of genes and traits, respectively (Table 1). Unlike a trait, a disposition is not a directly observable property, rather it is a potential property of the system. For example, a protein may have the evolutionary disposition of instability where its structure may change dramatically when mutated. Designing the dispositions of the evotype is a challenge fundamental to engineering biology.

For all but the very simplest biosystems, it is impractical to enumerate every potential evolutionary disposition, just as it is impossible to consider every trait of the phenotype. Instead, an appropriate sample of the evotype must be used for the purpose at hand, just as samples of traits are used when describing the phenotype. How we take this sample, and thus the scope of the evotype covered, should be determined by knowledge of the design type, its intended function and the context in which it will be used. This could include the size of population, environment and required number of generations over which the system must operate reliably.

Broadly speaking, we may wish to seek one of two goals when designing the evotype: the first is that of evolutionary stability, where a system changes its function as little as possible, as it evolves during use; the second is specific evolvability, where the system can easily evolve new phenotypes of a specific class (i.e., the classes of function specified by the engineer) or adapt to changes in the environment (i.e., continuing to produce a desired chemical product). Specific evolvability requires an element of robustness: core functions of the phenotype must remain unperturbed throughout sequence space so that new phenotypes can be explored. This is analogous to natural evolvability^21^, where the ability to generate novel phenotypes alone is insufficient, as they must also be adaptive. It also relates to the concept of plasticity, which is the ability to generate new features without total loss of function^22^. The relationship between robustness and evolvability in natural evolution has been explored in detail in prior literature^23^. For example, a genetic circuit may have been specified to produce an OR logic function in response to two input chemicals. That is, it expresses an output protein if either one of the two input chemicals are present. A population with an evolutionarily stable version of this circuit is likely to maintain an OR function during use. A specifically evolvable version of the circuit on the other hand might be designed to readily produce other logic functions when evolved (e.g., AND, NOR, and NOT), without simply destroying existing functionality or causing lethality to the host cell.

Whether evolutionary stability or specific evolvability is the goal, it can be achieved through engineering genetic variation, the production of function from genotype and both natural and artificial selection. How these processes interact to constrain and bias evolution can be understood by describing a landscape surrounding the design type in sequence space. We term this landscape the evotype, which extends and generalises the fitness landscape concept as applied to natural systems^24^ by accounting for the roles of variation, production of function and selection (both natural and artificial) in engineered biosystems. The bioengineer’s goal is to sculpt the evotype’s landscape to their specification, to ensure it has a structure in line with their requirements.

## Engineering genetic variation

The processes of genetic mutation and recombination are often considered to be random in nature. However, the types of variation that can occur and their associated probabilities are often heavily biased and constrained by the biochemistry of the biosystem itself, limiting the paths accessible to evolution^25^. As these constraints are partly determined by the biosystems genotype, genetic variation is something that can, in theory, be genetically engineered. For example, not all point mutations are equally likely; transversions and transitions differ in their likelihoods^26^, and methylation^27^, genomic context^28^ and species^29^ all influence local and global mutation rates. Furthermore, algorithmic mutations^30^ may occur. These are mutations that result in changes of several nucleotides in one event (thus, an algorithm can describe the change) and can be thought of as shortcuts through sequence space (Fig. 1b, left). The likelihood of an algorithmic mutation may be much greater than the summed likelihoods of the equivalent sequence of individual point mutations. For example, the chance of an insertion of the two-base motif ‘AC’ into a tandem repeat region due to slipped-strand mispairing may be more likely than two insertion events of ‘A’ and ‘C’ occurring independently^31^. Recombination^32^ and mobile genetic elements^33^ are other examples of biological processes capable of producing algorithmic mutations.

Sequence space is therefore not explored in a uniformly random way, even discounting for the role of selection. Instead, the paths evolution can take are determined by the ‘variation operator set’, which defines all the different point and algorithmic mutations that can occur in the system. Each variation operator in this set has an associated probability distribution that represents the likelihood of arriving at a given sequence from another (i.e., by this operator acting on the design type). The distributions of the variation operator set combine to produce the ‘variation probability distribution’. This describes the chance of arriving at any given sequence from the design type due to all the biochemical and physical processes capable of causing genetic variation that are present in the system (Fig. 1b, right). The variation operator set defines the rate and the likely directions in sequence space a design will explore during evolution. As a design type evolves, the variation probability distribution changes, as further dispositions become available.

The variation operator set depends on the specifics of the biosystem being engineered and the set to be applied in practice is dependent on available knowledge of the system. For example, the variation operator set of a design-type biosystem may be said to include transition mutations, transversion mutations and recombinations, each associated with a unique probability that varies across the design type’s sequence. A sample population can be generated by applying the operator set to the design type. This population, with the design type at its centre, may be named a quasispecies, as is used for the related concept in viral evolution^34^.

The variation probability distribution can be considered at all stages of the design process: from specifying mutation rates of specific parts, designing new biochemical mechanisms capable of specific forms of genetic variation and thinking of genetic variation as a feature of a system that can be designed and built. Such integration would allow for global and local mutation rates to be specified as part of the design and standardised mutation rates could even be listed in part datasheets^35^. It is likely that improvements in the prediction of mutation probabilities will be made with the increasing availability of sequence data and associated computational methods. Furthermore, some design rules for influencing local genetic variability are already known (e.g., avoiding the reusing of parts and repetitive sequences to reduce homologous recombination and indel mutations)^36–38^, and global mutation rates can also be rationally engineered and manipulated^12,39^.

A large toolkit for controlling genetic variation has already been created by bioengineers, which could be used to improve evolutionary stability or increase specific evolvability (i.e., the ability of the biosystem’s evolution to be directed as the designer intended). New tools will doubtless be developed from the diverse mechanisms that generate genetic variation in nature. The variation probability distribution of the design type can be modified by either adding or removing variation operators (e.g., by adding or removing DNA modifying enzymes) or by modulating existing operators in the system across the genotype. This may be through altering DNA sequence properties (e.g., avoiding simple sequence repeats to reduce the chance of indels through slipped-strand mispairing^36^). Variation operators can be highly targeted like the DNA methylation of specific bases to increase likelihood of mutation through spontaneous deamination^27^ or may have a global effect such as the removal of error-prone polymerases from a host organism^40^. Orthogonal mutation systems that modulate genetic variation of a specific plasmid or region of DNA can be used to overcome genomic error thresholds, increasing the potential for directed evolution^41^.

Larger-scale genetic variation can be achieved through mechanisms such as site-specific recombination, which can be used for inserting, removing, duplicating, inverting or shuffling large segments of DNA, exemplified by the SCRaMbLE system used in the synthetic yeast Sc2.0^42^. Finally, acquisition of foreign DNA either from other organisms in the population through sex, horizontal gene transfer or from free oligonucleotides in the environment^12^ may also be engineered. The recombinant approaches of genetic engineering can be thought of as a highly orchestrated form of horizontal gene transfer, which is also increasingly being acknowledged as a source of innovation in natural evolution. For example, it is a major mechanism used by bacteria to acquire antibiotic resistance^43^. As with sexual recombination, it enables large jumps through sequence space. This increases the breadth of search and potentially enables the crossing of valleys in the evolutionary landscape to access peaks that would otherwise be inaccessible.

By combining these and other biochemical tools, it may eventually be possible to precisely design the variation operator set to produce complex combinations of genetic variation. For example, the variation operator set of a genetic circuit may be engineered by avoiding repeated parts (removing the homologous recombination operator), using a host with a high-fidelity DNA polymerase (globally reducing probability of point mutations), and by incorporating DNA recombination sites (adding an operator for specific DNA recombination, perhaps to be used for future directed evolution). Table 2 provides some examples of methods for controlling variation operators that have been developed so far.

**Table 1:** Role of genotype, phenotype and evotype when describing biological systems.

	Genotype	Phenotype	Evotype
Describes…	Informational/hereditary properties	Physical/environmental properties	Evolutionary properties
Is a set of…	Genes/sequences	Traits	Dispositions
Pig example	Hox gene	Four legs	Chance of evolving wings
Protein example	Codon	Structural stability under temperature^a^	Structural stability under mutation^b^
Biosensor example	Genetic circuit sequence	Sensor sensitivity/specificity, Input-output function, Fluorescent output, etc.	Probability of: total failure, sensitivity loss, function change, etc.

^a^For example, strengthened hydrophobic interactions in the interiors of thermostable proteins^101^.

^b^For example, robustness of the genetic code to amino acid mutations due to synonymous codons^102^.

## Engineering the production of function

Genotypes produce phenotypes via the processes of gene expression, growth and development. However, due to the constraints and biases of these processes^44^, phenotypes are not necessarily distributed evenly throughout sequence space and not all conceivable phenotypes may be possible. Furthermore, in the same way that multiple genotypes can achieve the same phenotype, a population of cells with identical genotypes can also potentially display many different phenotypes due to the stochastic nature of the underlying processes^45^ or their sensitivity to environmental fluctuations (e.g., displaying chaotic dynamics^46^). Many systems have shown similar properties in the structure of their mapping from genotype to phenotype. Namely, redundancy (there are many more genotypes than phenotypes) and bias (a small fraction of phenotypes are over-represented). This has been shown both through simulation^47^ and experimentally in RNA and protein structures^47^, and DNA-binding sites^48^. How these principles apply to more complex biosystems is a major challenge due to their vast genotype spaces. Nevertheless, there will be a statistical structure in the mapping of genotype to phenotype. If this structure is sufficiently well understood, it could offer a powerful way of engineering the evotype.

Engineered biosystems have phenotypic traits that influence reproductive fitness and traits that influence ‘function’—the behaviour or properties specified by the designer (although these are not mutually exclusive). The structure of the mapping from genotype space to function space is therefore a key part of the evotype. Function space may be discrete or it may be continuous (Fig. 1c). In complex systems, such as biochemical networks, even continuous variation of parameters during evolution can result in the production of identical functions or cause phase changes where qualitatively different functions arise^49^. Designed functions could be literal mathematical functions, physical characteristics such as colour or size, or combinations of several properties.

Any designed system has a degree of ‘utility’—the extent to which the system fulfils the specified function. The sole goal of a traditional engineering design process is to maximise the utility of the design type. However, the topology of the function landscape surrounding the design type is also important. It may be rugged and highly variable with the function rapidly changing across sequence space or it may be smooth and have large neutral regions where function changes little or remains constant. Whether the goal is to evolve novel functions or to tune the parameters of an existing one, these properties are a key design consideration: what is the functional range to be covered by the design type’s evotype? Should the variation be large for increased evolvability, or limited, for evolutionary stability? Most likely, the function landscape should be smooth and predictable, but how is this best achieved? Which regions of function space must be avoided, and which can be tolerated? For example, it may be necessary to reduce irrelevant or harmful functions as much as possible in a diagnostic application where regions of function space cause false negatives, whereas regions causing false positives can be tolerated.

Designs may have identical functionality but occupy regions of function space with very different topological properties. If a system is designed without considering its surrounding function landscape, a design with an undesirable evotype may be a likely outcome. Systems with identical phenotypes, yet differing function landscapes, were demonstrated by Schaerli et al.^50^, who designed two genetic circuits, both with the same strip-generating function. It was found that each produced a different spectrum of new phenotypes when mutated due to differences in the regulatory mechanisms used^50^. We are only just beginning to understand what influences the structure of the mapping from genotype to phenotype. However, there are some general principles, which seem to hold across scales and contexts. Fortunately, many of these principles are already familiar to engineering (Table 3).

### Prevalent phenotypes

Phenotypes that are more prevalent in sequence space can be both more robust (as they are more likely to be in genotype networks sharing the same phenotype), and more evolvable (as this allows a wider search of genotype space, increasing access to more novel phenotypes)^23^. Therefore, choosing prevalent components may aid both evolutionary stability and specific evolvability. For example, if designing a protein, the codon chosen may influence evolvability: for a leucine residue, if UAA is chosen, its 1-mutant neighbourhood has a lower prevalence for leucine than any other codon (two vs. four, respectively). Therefore, UAA may have lower evolutionary stability but higher specific evolvability than other codons (as it is able to generate a wider range of non-polar amino acids). Remapping the genetic code itself has been suggested as a way of altering its evolvability^51^. Other examples of applying phenotypic prevalence include choosing RNA or protein structures that are highly represented in sequence space^52,53^. An interesting question is how the phenotypic prevalence of a system’s parts relates to the overall phenotype compared to higher-order properties? Is the robustness of a genetic circuit’s parts or its network topology a greater determinant of its overall robustness?

### Redundancy

Redundancy is used in classical engineering and by evolution. It can add robustness by allowing variation of parts of a system without overall loss of function and can aid in evolvability by enabling redundant parts to mutate and thus explore new regions of function space. This can be seen in serial homology, where repeated parts such as the limbs or teeth enable evolution of specialised functions^54^, in gene duplications^55^ and in the scale-free structure of genetic networks where most nodes can be removed without altering the overall function^56^. It is noteworthy that redundant parts may either be repeats of the same element or different elements that can produce the same function (often termed degeneracy^57^).

### Modularity, regularity and hierarchy

The organisational properties of a biosystem are a major influence on its evotype. These can be summarised by modularity, regularity and hierarchy^58^. Modularity is the division of a system into subsystems (modules), where each has a high degree of internal connectivity, but little interdependency between subsystems. Examples of this can be seen in the connectivity of protein and regulatory networks, in RNA structures and in limb development^59^. Regularity is the use of patterns, repetitions and symmetries (e.g., serial homology and animal body plans). Hierarchy is the recursive arrangement of a system into subsystems (that are themselves composed of subsystems, etc.)^60^. For example, an organism is composed of organs, which is in turn composed of tissues, cells, etc. Hierarchy is also seen in gene regulatory networks. For example, only nine proteins regulate half of all genes of *Escherichia coli*^41^.

These principles are distinct but often work together. For example, identical modules are often repeated in regular patterns and modules are arranged in a hierarchical structure. These principles may each promote evolvability in different ways. Modularity allows parts of a system to mutate and change function with a reduced negative impact on the rest of the system. Efforts to improve the modularity of genetic systems have been made by synthetic biologists by standardising and increasing orthogonality between parts. Regularity reduces the information required to describe the system (e.g., its genotype), essentially reducing the size of the search space. Hierarchy allows the progressive increase in the complexity of a system from the bottom up^60^. Although the widespread use of these principles in both biology and technology clearly demonstrates their importance, how and where these principles should be applied is context specific. This can be illustrated with an imagined example.

Consider two biosensor circuits that each use red, green and blue (RGB) fluorescent proteins to produce a white output. In circuit A, the overall output of RGB should be as high as possible when the input is positive (e.g., high sensitivity is required), the whiteness of the signal is less critical. In circuit B, it is important that the positive signal remains precisely white (e.g., other colours represent other input conditions) and the overall output level is less critical. Circuit A would benefit from a modular arrangement of RGB, because a mutation in any one of these genes does not affect the other two, thus reducing the impact on overall output. However, for circuit B, a less modular design would be preferable: although a mutation would have triple the effect on overall output, all colours would be impacted equally conserving the overall hue. The nature of the design problem therefore relates to how modularity should be used. In fact, Kashtan and Alon^61^ showed that modular architectures evolve in gene regulatory networks, in response to modular environmental selection pressures, and themselves prove to be more evolvable^61^. Similar relationships between how hierarchy and regularity should relate to the design problem, no doubt, exist. However, we are far from having design principles for their application, in particular for more complex problems.

### Environmental robustness

Principles that improve robustness to environmental change or noise may also improve robustness to genetic change. For example, if a genetic circuit is robust to noise in the concentration of a regulatory protein, it may also be more robust to mutations that change the promoter’s expression level^62^. Similarly, proteins that are more thermodynamically stable may also be more evolvable^63^. Systems could be buffered against environmental and genetic perturbations through the use of a negative feedback^64–66^, tunable genetic parts^67^, stringent multi-level regulation^68^ or the application of other control engineering principles^69^.

### Designing parameter space

The structure of the parameter space of a system plays a large role in how function changes under genetic variation. If the behaviour of a system can be modelled or inferred against the variation of key parameters, this can provide information about which functions may be accessible and most likely throughout sequence space. For example, by modelling the regulatory mechanisms of two genetic circuits, Schaerli et al.^50^ explain why they produce different distributions of functions when subjected to point mutations. Similarly, with a simple mathematical model of equilibrium binding, Mayo et al.^22^ showed that the *cis*-regulatory region of the *lac* operon in *E. coli* is incapable of accessing some input functions via point mutation. Parameter spaces are analogous to the morpho-spaces of evolutionary-developmental biology, which provide constraints on organismal form^70^.

### Other principles

System-specific principles may also provide design rules for the evotype. For example, RNA gene regulation may be less evolvable than transcriptional regulation^71^ and so could determine whether the regulation is applied at the transcriptional or translational level. Physical and chemical processes of self-organisation may even be able to reduce a function’s dependency on the genotype. Perhaps ideas from developmental biology and morphogenesis could be recast into engineering terms, such as concepts from the theory of facilitated variation^72^, in particular as bioengineering progresses to multicellular organisms. Metaheuristic design approaches will also no doubt become an increasingly powerful tool: machine learning approaches may be able to predict the evolvability of biological networks^73^ and genetic algorithms have been used to evolve more robust genetic networks in silico^74^.

## Engineering natural and artificial selection

Selection is the force that gives the otherwise random (but constrained) processes of genetic variation a ‘direction’ by driving a population up the slopes of the adaptive landscape^75^. Uniquely, an engineered biosystem is a result of two forms of selection: natural selection and the design process. Natural selection acts on reproductive fitness of the biosystem and the design process can be thought of as a sophisticated form of artificial selection acting on its utility. Fitness and utility both form part of the evotype and understanding the interplay between these two processes is critical for effective evotype design, as there is often a tension between the two (Fig. 1d). If fitness and utility are uncorrelated, then natural selection is likely to undo the work of the engineer. However, if fitness and utility are highly correlated, then natural selection will also increase utility^76^. For example, one might design a cell in a bioreactor or a plant crop to produce a chemical product. Perhaps, it uses control circuitry to maintain optimal metabolic fluxes to maximise yield in fluctuating environmental conditions, thus resulting in high utility. However, this will inevitably have a fitness effect on the organism (e.g., due to the metabolic burden of the circuit or toxicity of the product) and, thus, natural selection will favour mutants where this functionality is repressed. It should be noted that natural selection here is meant as the process that acts on the reproductive ability of the biosystem. Neither the environment nor biosystem need to be natural (e.g., the organisms could be engineered to make use of non-canonical amino acids and grown within a bioreactor). The critical distinction is that natural selection acts on survival of the biosystem without the input of the engineer.

The aim of a bioengineer then, is to maximise ‘fitneity’—defined as a function that combines the derivatives of utility and fitness (Fig. 1d). Ultimately, evotype engineering is controlling how fitneity changes throughout sequence space: it is the sculpting of the fitneity landscape. Exactly what form the fitneity function should take and the best way to mathematically describe the fitneity landscape to effectively capture the interaction between these two forms of selection are not yet clear. Defining, modelling and characterising the fitneity landscapes of designed biological systems is a future avenue of research ripe with potential. Nevertheless, the concept can already help in thinking about how to improve the fitneity of designs on an intuitive level.

To design for evolutionary stability, it is sufficient to limit or neutralise the impact of natural selection. This can be achieved in one of the following three ways. First, the fitness of the design type and its immediate neighbours can be increased to create a local peak or plateau. This could be done through adaptive evolution^77,78^ after the design phase, reducing or dynamically controlling burden^65^, or by reducing toxicity of the associated function. Second, the fitness of neighbouring genotypes can be decreased to flatten the surrounding fitness landscape, e.g., by using organisms with a reduced genome that may be less fit than wild-type organisms^79^, but with freed-up metabolic resources^80^. Third, the utility landscape can be flattened so that even if there is a natural selection pressure away from the design type, it is less likely to impact the design’s function. Approaches for doing this have been outlined in the previous section. To ensure a specifically evolvable evotype, fitness and utility must correlate: both fitness and utility must slope in the same direction. An engineer could do this by coupling function to survival, perhaps through a toxin–anti-toxin system^81^ or by coupling function to growth (e.g., by having the product of a system aid in metabolism of an energy source). Alternatively, an artificial environmental pressure, such as repeated screening, could be used to ensure utility and fitness correlate. Common methods to engineer selection are shown in Table 4.

## Toward evotype engineering

The evotype is a new way to think about the properties of engineered biosystems and how they relate to each other (Table 3). It is a framework for thinking about an important but often overlooked property: the role the biosystem itself plays in its future evolution. This is especially critical due to the impact of an intervention (e.g., a new mutational method) being closely linked to the composition of the system itself. For example, in a simple case, an identical protein could be encoded by very different sequences and so be impacted by a targeted mutating element in different ways. This is quite different to how engineers normally view systems. As engineered biosystems are the result of both human creativity and natural adaptation, a holistic consideration of both the roles of design and evolution is necessary. The evotype helps us do this by explicitly considering the intertwined effects that genetic variation, production of function and multiple forms of selection will have on a design (Fig. 1).

**Table 2:** Methods to engineer genetic variation.

Mechanism	Type of variation	Stability	Evolvability	Examples
Site-specific recombination	Inversions Deletions Duplications Rearrangement	Remove host recombinases	Targeted recombination systems	CRISPR-recombinase^103^, SCRAMBLE^42^, De novo recombination sites^104^
Homologous recombination	Rearrangement Deletions Duplications	Remove host recombinases Avoid repeated sequences	Inducible recombination Design in repeated sequences	Heritable recombination system^105^ EFM calculator^36^, Automated design of non-repetitive parts^38,106^
Mobile genetic elements	Rearrangement	Remove transposons	Transposon mutagenesis	Abolishment of mobile genetic elements^82,107^, CRISPR-controlled insertion sequences^108^, In vivo transposon mutagenesis^109^
DNA polymerase fidelity	Global point mutation rate	Eliminate error-prone polymerases	Mutator strains	Low mutation *E. coli*^40^, Xl1-Red mutator strain^110^, Mutagenesis plasmids^111^
	Local point mutation rate	–	Targeted DNA polymerase	CRISPR-DNA polymerase^112^
	Plasmid-specific mutation rate	–	Orthogonal mutation plasmids	Orthorep^41^, Pol 1 mutagenesis system^113^
Slipped-strand mispairing	Targeted small indels	Avoid simple sequence repeats	Employ simple sequence repeats	EFM calculator^36^
DNA methylation	Point mutation	Remove host DNA methyltransferase	Targeted methylation	CRISPR-methylation^114^
Cytosine deamination	Point mutation	–	Targeted deamination	CRISPR-deamination^115^

**Table 3:** Methods to engineer the production of function.

Principle	Stability	Evolvability	Examples
Prevalence	Use prevalent phenotypes	Use prevalent phenotypes	Designable protein structures^53^
Redundancy	Use multiple copies of genes/constructs	Use multiple copies of genes/constructs	High gene-copy system for *E. coli*^116^
	Scale-free networks^117^	Scale-free networks	–
Modularity	Insulate genetic parts	Insulate function	Ribozyme insulators^94^, Insulated genetic landing pads^118^
	Use orthogonal systems	Use orthogonal systems	T7 RNA polymerase^119^
	Spatial/temporal separation	Spatial/temporal separation	Microbial consortia^120^, Targeting to a cell cycle stage, specific cell type or organelle^121,122^
Regularity	Standardise parts across system	Standardise parts across system	Standardised architecture of the Yeast 2.0 genome^123^
Hierarchy	Co-control related functions	Co-control related functions	–
	Use networks with wide, shallow hierarchies	Use networks with wide, shallow hierarchies	–
Environmental robustness	Feedback control	Feedback control	Feedback control of transcription and translation^124^
	Use larger tolerances for parts	Use larger tolerances for parts	Maximise dynamic range of genetic logic gates^94^
Designing parameter space	Design a constrained parameter space	Design a diverse parameter space	Modelling phenotype distributions of genetic circuits^50^

**Table 4:** Methods to engineer natural and artificial selection.

Principle	Stability	Evolvability	Examples
Increase design-type fitness	Evolution for fitness after design phase	–	Adaptive evolution of recoded *E. coli*^77^, Adaptive evolution of refactored phage genome^78^
	Reduce metabolic burden	–	Modelling ribosome allocation to reduce burden^125^, Characterising burden of genetic parts^126^
	Dynamic control of burden	–	Burden-driven feedback control in *E. coli*^65^
	Reduce toxicity	–	mRNA toxicity^127^
Decrease neighbouring fitness	Minimised chassis organism	–	*Pseudomonas* 2.0^80^
Correlate fitness and utility	Couple function to reproduction	Couple function to reproduction	Phage-assisted continuous evolution^10^, Compartmentalized partnered replication^128^
	Couple function to survival	Couple function to reproduction	Toxin/antitoxin systems^81^
	Artificial selection pressure	Artificial selection pressure	FACS screening^129^, Ribosome display^109^, Phage display^130^

We can now design and build genotypes with great precision, but we must account for the inevitable processes of genetic variation that will follow. The statistical structure of variation is unique to each biosystem and something we have control over. Yet, understanding the details of genetic variation is insufficient if we do not understand how this will manifest in changes of the designed function of the biosystem as well. Even a system with low mutation rates can be evolutionarily unstable if function changes wildly with small sequence alterations. Similarly, directed evolution will not be successful, despite the mutation strategy, if desired functions are simply not accessible from the starting point. If the biosystem’s utility (i.e., its success as a design) and its fitness (i.e., its success as a biological replicator) are at odds, well-designed dispositions for variation or function might not save the design from the pressure of natural selection. This must also be understood as a conflict between utility and fitness landscapes across sequence space surrounding the original design type. It is clear then that all three of the aspects of the evotype must be considered together and all offer significant scope for engineering. For instance, imagine a large genetic circuit that places an unavoidably high metabolic burden on the host cell. If it is crucial that the function of the circuit is maintained over long periods of time, then redundancy could be used to accommodate unavoidable mutations. However, if the dent to reproductive fitness is severe, this may still not be enough. Therefore, combining redundancy in the design with a hyper-stable host cell (e.g., one where all mobile genetic elements have been deleted and efficient DNA repair mechanisms are present^82^) might be the only way to achieve the desired goal for the system.

Designing biosystems with evolution in mind is a vital step towards a more complete engineering theory of biology. However, to be practical, supporting tools must exist that can provide key information regarding the genetic variation, genotype-function map and selective pressures within a biosystem. Advances in sequencing offer a means to quantitatively measure millions of genotypes in parallel^83^ and when combined with high-throughput techniques, such as fluorescence-activated cell sorting, make it possible to infer simplified genotype-function maps^84,85^. The local function landscapes of the green fluorescent protein^86^ and transcription factor-binding sites^48^ have already been characterised experimentally with such methods. Detailed measurements of fitness in large populations of cells are also possible^87–89^. By combining sequencing with expression and growth measurements, genetic variation, function and fitness could be characterised simultaneously to provide a complete picture of the evotype.

Even so, the vastness of evotype landscapes and the need for functions calculated from many outputs of a system mean that new methods with greater throughputs are also necessary^85,90^. There is a particular need for methods able to measure many characteristics of each cell simultaneously (e.g., via automated high-content microscopy^91^ or high-throughput Raman spectroscopy^92^). Parallel to these experimental methods, a promising direction to bypass the need to directly measure these properties are the development of sufficiently comprehensive computational models (e.g., encompassing whole cells^93^) to allow for a mechanistic understanding of the biases in processes related to variation and reproductive rate. In these cases, if they are sufficiently accurate, the evotype could be predicted and used within computer-aided design workflows^94^ to reduce the need to physically build every possible design.

Nevertheless, for systems of even moderate complexity, the evotype landscapes are much too vast to be exhaustively characterised or even modelled. It will therefore be of great importance to understand how they should be sampled^95^, how large a region of the landscape needs to be characterised and to what extent local landscape properties can be extrapolated. The use of machine learning is another method that holds great promise for increasing the ability to estimate the evotype landscape, albeit at the cost of mechanistic knowledge of the system.

Epistasis poses a particular challenge to the prediction and the engineering of evotypes, as it means even a small number of mutations can have large effects that are difficult to predict^96^. In these cases, the engineer may have little choice but to limit the likelihood of such point mutations occurring and to use more constrained variation operators that act at a structural level (thus, smoothing and reducing the dimensionality of the evolutionary search space), such as the recombination of insulated parts. However, some evidence suggests that biologically relevant fitness landscapes may in fact occupy a low dimension of total sequence space^97^. This offers hope that as least in some contexts, evotypes can be characterised, predicted, and designed with some accuracy. The predictability of evolution is one of the most important and challenging unanswered questions in the study of natural biological systems^98^. However, engineers have the advantage of being able to design systems to suit their needs. One way to do this is to design systems to maximise forms of evolution that can be predicted and minimise those that cannot.

**Fig. 2:**
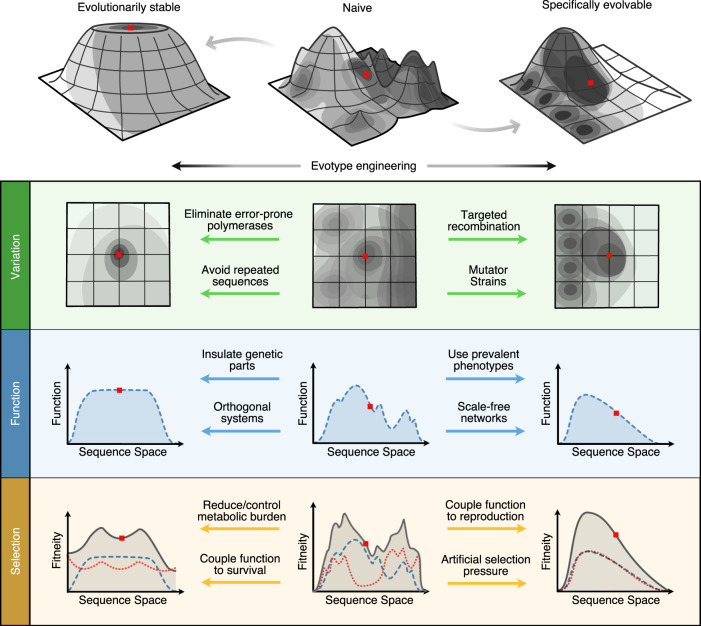
Engineering evotypes by sculpting their landscapes. Different biosystem designs may share the same phenotype but have very different evotypes (top row). Rational engineering approaches could be used to transform a naive design (middle column), where evotype has not been considered, into either evolutionarily stable (left column) or specifically evolvable (right column) evotypes, which are characterised by their fitneity landscapes. Bioengineers can sculpt the evotype by modifying three major factors: genetic variation, production of function and selection. Genetic variation (green row): in a naive design, a mixture of variation operators may be in play. This might create a system that can reach many different regions of sequence space. It could be made more stable by reducing global mutation rates (e.g., host strain engineering) or by removing homologous regions to reduce the chance of recombination. Conversely, a naive design might be made more evolvable by increasing mutation rates in focused areas of sequence space (e.g., via methylation) and incorporating site-specific recombination or gene shuffling (e.g., the SCRaMbLE system). Function (blue row): a naive design may have high utility; however, if its function changes rapidly and chaotically across sequence space, it may be inherently unstable. A robust evotype has large neutral regions in function space. Conversely, a design can be made more evolvable if it can access a large range of new phenotypes, of a specific class (e.g., produce a colour) and the landscape may be smoothed (e.g., through removing crosstalk between features) and thus made amenable to evolutionary search. Production of function may be engineered by using prevalent phenotypes, designing in redundancy, modularity, regularity and hierarchy, increasing environmental robustness or by designing a system’s parameter space. Selection (orange row): if, as in the naive design, reproductive fitness (red dotted line) and utility (blue dashed line) are highly uncorrelated, then the design type may have a strong selection pressure acting against it and regions where both fitness and utility are maximised may be rare or non-existent; thus, high fitneity (grey solid line) may not be achievable. For a stable design, one might act to reduce the effects of natural selection through global increases in fitness (e.g., through reducing metabolic burden of a genetic circuit), by reducing toxicity of gene products or by reducing the fitness of neighbouring sequences (e.g., using minimised chassis organisms). A naive design can be made more evolvable by closely correlating fitness and utility (e.g., through coupling function to reproduction). This means natural selection will act to drive up the utility of the design: the precise goal of a directed evolution experiment.

In addition to characterising evotypes, tools for bioengineers to directly sculpt their landscapes must also be available (Fig. 2). Here we have touched upon the numerous repurposed biomolecular components that can alter the types of possible variation (Table 2). However, there is a spectacular diversity of molecular machines dedicated to manipulating genetic information in the natural world, suggesting a need for an even larger toolkit to precisely modify genetic variation as needed. Likewise, principles for constraining and biasing the production of function (Table 3) and for controlling selection pressures (Table 4) have been suggested, but they are still poorly understood and have barely been applied rigorously in an engineering setting. It is also important to recognise that engineers may not always be in a position to influence all aspects of the evotype. For example, if function and survival cannot be linked effectively (i.e., selection cannot be engineered in a necessary way), then only variation and/or function are available to the designer. Thus, the practical constraints of a given design problem will often determine which evolutionary design methods are available or are most appropriate.

We have been careful throughout this work to clarify the differences between natural evolution based on natural selection (i.e., fitness) and artificial evolution based on our own forms of selection. However, there are cases where their differences become blurred. For example, when an engineered biological system has reproductive success coupled to utility, is this system naturally or artificially evolving? Much of these confusions stem from semantics of the framework used to interpret the system and here we have explicitly shown that in engineered biology the term “evolution” is almost always a mix of both natural and artificial contributions. Moving forward, ensuring that the terminology we use is consistent and clear will be crucial for supporting the robust development of an engineering theory encompassing all forms of evolution.

Traditional engineering disciplines have developed various methods that are somewhat analogous to some of the evolutionary principles outlined in this work. For example, the use of modular parts and hierarchical designs, fault tolerance, factors of safety and redundancy to improve robustness, the reuse of parts and building in tolerance to variation. However, there are clearly differences in exactly how and where these principles are applied in biological vs. engineered systems. A deeper understanding of the relationship between engineering principles and their evolutionary counterparts is needed. Better awareness of how evolution applies these principles will improve our ability to engineer all types of complex systems, in particular those that evolve.

It is also crucial to recognise that evolution is not the only challenge faced when engineering biology. Unlike many of the substrates we commonly build with, biology is highly complex even at its simplest level (e.g., single cells), with changing and growing components that can deform and exhibit intricate phase transitions due to the many nonlinear interactions present. Although our focus here has been solely on evolution, an ability to effectively engineer living systems will require a holistic approach that considers and integrates these other aspects and goes far beyond current working practices.

Another area of growing importance in biological engineering is the development and adoption of standards to facilitate improved exchange and reuse of engineered biological parts and systems^99^, as well as data associated with these^100^. Standards are also pervasive in natural biology with an example being the use of a (mostly) common genetic code that aids the exchange and reuse of genetic material in the wild. To date, evolution has not featured prominently in standardisation efforts, but could be key to the collection of information about biological parts and systems (e.g., in terms of mutation rates, operator sets, function sets, selection strengths and pressures, evolutionary stability, etc.), which will support the future engineering of evolution.

The lens of engineering offers a fresh perspective on evolutionary theory. It is also a new way of thinking about what it is that engineers do and what the design process is in the context of bioengineering. The concept of the evotype, with some modifications, may also find use in evolutionary science, where it offers a framework for considering the mechanistic constraints of evolution and a way of talking about the evolutionary characteristics of organisms. It may also be applied beyond biological engineering fields to create new self-adaptive technologies. In that context, the framework could be applied to ask how we design technologies to evolve and not just how to engineer systems that already do.
